# Feedback Training Improves Compliance with Sternal Precaution Guidelines during Functional Mobility: Implications for Optimizing Recovery in Older Patients after Median Sternotomy

**DOI:** 10.1155/2021/8889502

**Published:** 2021-01-25

**Authors:** Ansel LaPier, Kimberly Cleary

**Affiliations:** Physical Therapy Department, Eastern Washington University, Spokane, Washington, USA

## Abstract

Patients often need to use their arms to assist with functional activities, but after open heart surgery, pushing with the arms is limited to <10 lb (4.5 kg) to help minimize force across the healing sternum. The main purposes of this study were to determine if older patients (>60 years old) (1) accurately estimated upper extremity (UE) weight bearing force of 10 lb or less and (2) if feedback training improved their ability to limit UE force and pectoralis major muscle contraction during functional activities. An instrumented walker was used to measure UE weight bearing force, and electromyography was used to measure pectoralis major muscle activity simultaneously during 4 functional mobility tasks. After baseline testing, healthy older subjects (*n* = 30) completed a brief session of visual and auditory concurrent feedback training. Results showed that the self-selected UE force was >10 lb for all tasks (20.0-39.7 lb [9.1-18.0 kg]), but after feedback training, it was significantly reduced (10.6-21.3 lb [4.8-9.7 kg]). During most trials (92%), study participants used >12 lb (5.5 kg) of arm weight bearing force. Pectoralis major muscle peak electromyography activity was <23% of maximal voluntary isometric contraction and was reduced (9.8-14.9%) after feedback training. Older patients may not be able to accurately estimate UE arm force used during weight bearing activities, and visual and auditory feedback improves accuracy and also modulation of pectoralis major muscle activation. Results suggest that an instrumented walker and feedback training could be clinically useful for older patients recovering from open heart surgery.

## 1. Introduction

To access the heart, median sternotomy is performed during a variety of different surgeries such as coronary artery bypass, heart valve replacement, heart transplantation, and thoracic trauma repairs. The procedure entails making a midline skin incision from the sternal notch to the xiphoid process, dividing the subcutaneous tissue and fascia, and separating the sternum with retractors. Finally, a variety of wiring techniques are used to reunite the sternal halves after surgery completion [1]. Because it allows for optimal visualization and access to the heart and mediastinum, median sternotomy is frequently used during cardiac surgery despite the development of less invasive techniques [2].

Median sternotomy is associated with a variety of complications including superficial and deep wound infections, bony nonunion/sternal instability, and sternal dehiscence [3–5]. Many pre-, peri-, and postoperative factors increase a patient's risk for developing these complications. Previous research has identified many of these risk factors including obesity, female gender, diabetes, history of smoking, disability, intraoperative blood loss, redo sternotomy, bilateral internal mammary artery grafting, prolonged mechanical ventilation, cardiopulmonary bypass, and surgical time [6, 7]. In order to avoid many of the complications associated with median sternotomy, upper extremity (UE) activity limitations are prescribed to minimize postsurgical stress across the healing sternal halves [5, 7–9].

One of the most common and significant sternal precautions is the restriction of weighted UE movements to 10 lb (4.5 kg) or less. This strict parameter directly limits UE use in many daily tasks such as carrying groceries, unloading clothes from a washing machine, or even just getting out of bed. The rationale for these restrictions is to promote bone healing by minimizing sheer and distractive forces across the sternum and motion between the sternal edges [4, 8, 9]. As one might expect, it is difficult to function independently with such severe limitations in place for daily tasks, especially for older adults who make up the majority of patients undergoing cardiac surgery [10, 11]. Restricting UE use is particularly problematic for patients who need assistance sitting down or standing up from a chair and/or need to use a walker for ambulation. This loss of functional independence can contribute to increased time in the hospital after surgery and a greater need for assistance and rehabilitation after hospitalization [12, 13]. These limitations on UE use impair patient function during mobility tasks and activities of daily living immediately after hospital discharge and sometimes remain even 6-12 months after surgery [11, 14, 15]. Outpatient cardiac rehabilitation has well-known benefits with regard to improving patient functional capacity, but referral and attendance are low [16, 17]. Paradoxically, patients with low functional capacity and/or frailty are less likely to participate in cardiac rehabilitation after hospitalization than their higher functioning counterparts [18, 19]. Therefore, teaching patients appropriate UE use is important for optimal recovery and functional independence following cardiac surgery.

To date, few studies have examined weight bearing force through the UE or pectoralis major (PM) muscle activation during functional mobility tasks. Previous studies have found that force when using a single arm to assist with standing up from a bench was 27.5 lb (12.5 kg) [20] and while moving from side lying to sitting in a bed was 22.2 lb (10.1 kg) [21]. Limited information is available on UE force during ambulation with a walker in patients attempting to limit weight bearing to 10 lb (4.5 kg) or less. A previous study found that self-selected UE force when instructed to use less than 10 lb (4.5 kg) was 11.7-19.0 lb (5.3-8.6 kg) during ambulation with an assistive device and sit-stand transfers in a young (18-40 years old) cohort and that subjects used more than 12 lb (5.5 kg) of UE force during most trials (67%) [22]. In addition, PM muscle activity could provide a good estimation of force across the sternum since it is the primary muscle attached to this bone. Furthermore, the PM muscle has a lateral direction of pull across the sternum, which may be perceived as having the potential to separate the postoperatively rejoined sternal halves. Only a single study has previously measured PM muscle electromyography (EMG) activity during walker ambulation and sit-stand transfers and found that EMG values ranged from 3.0 to 9.2% of maximal voluntary isometric contractions (MVIC) [22]. Therefore, this study had multifold purposes and set out to examine functional mobility tasks in older adults to:

1. determine how accurately they can estimate using ≤10 lb (4.5 kg) of force through their UE.
2. assess the efficacy of feedback training (immediate and short-term skill retention) for improving the modulation of UE force and PM muscle EMG recruitment.
3. describe the degree of PM muscle activity.
4. evaluate the impact of lower extremity impairment on UE weight bearing force and PM muscle activity.
5. identify variables (balance, strength, frailty, and functional status) that influence UE weight bearing force.
6. determine if UE weight bearing force is symmetrical under normal conditions and with unilateral leg impairment.
7. determine if PM muscle activation is directly related to UE weight bearing force.

## 2. Methods

### 2.1. Participants

This prospective study used a within-subject design with repeated measures. Subjects (*n* = 30) were a convenience sample recruited from a university community via flyers posted around campus and sent electronically (email and text messages). Inclusion criteria were: (1) a healthy adult between the ages of 60 and 85 years, (2) able to complete Timed Up and Go Test in <14 sec, and (3) able to provide informed consent. Exclusion criteria were: (1) recent (<6 months) significant medical event (i.e., stroke and myocardial infarction), (2) pain exacerbated with UE movements/activities, and (3) any contraindication for exercise participation as outlined by the American College of Sports Medicine Guidelines for Exercise Testing [23]. This research project was reviewed and approved by the University's Institutional Review Board.

### 2.2. Force Measurements

Weight bearing force through the UE was measured using an instrumented walker. Force dynamometers (Jamar Smart, Performance Health, Chicago, IL) were wirelessly connected to tablets (Fire HD 10 Tablet, 1080p Full HD, Amazon, Seattle, WA) and interfaced with an application (Jamar Smart, Performance Health, Chicago, IL) that allowed continuous force data collection for up to 30 sec. The dynamometers were attached to the grip holds of a standard walker frame (Deluxe Two Button Folding Walker Drive, No. 10200-1, Drive Medical, Post Washington, NY) using platform attachments (Platform Walker/Crutch Attachment No. 10105-1, Drive Medical, Post Washington, NY) and 2.5 cm U-bolts. The front legs of the walker were replaced with wheeled legs (Universal 5” Walker Wheels, Drive Medical, Post Washington, NY) for the front wheeled walker trials. The front legs of the walker could also be replaced with extension legs (Tall Extension Legs, Drive Medical, Post Washington, NY) to accommodate subjects up to 200 cm tall. During sitting and standing trials, the instrumented walker was turned backward and placed behind a stool to simulate a chair with armrests. This configuration created a seat height of 46 cm and armrest height of 60 cm.

### 2.3. EMG Measurements

Surface EMG was used to measure bilateral activity of the PM muscles. Electrodes were placed 3.5 cm lateral to the anterior axillary line [24]. An additional ground electrode was secured to the study participant's left wrist. The electrodes had dual 1 × 10 mm, bipolar, silver-silver chloride surfaces, an interelectrode distance of 10 mm, and onsite preamplification with a gain of 1000. They were attached to an EMG data logger (DataLOG Multisensor System MWX8, Biometrics Ltd, Newport, UK) that employed a sampling frequency of 1000 Hz and a bandwidth of 20 to 450 Hz.

Surface EMG data obtained were processed and normalized. Raw EMG signals were analyzed (DataLOG Software, version 8.51, Biometrics Ltd., Newport, UK) and expressed as root-mean-square amplitude which is the square root of the average power of an EMG signal for a given period of time. A data capture window was set for each task between event markers placed during data collection. Normalization of the EMG activity was done by expressing data relative to a maximal voluntary isometric contraction (MVIC) of the PM muscle. This is a commonly accepted method to account for differences in EMG activity measurements based on muscle mass [22, 24, 25]. A palm press was performed with shoulders flexed 90 degrees bilaterally with the heel of the hands together and elbows flexed 20 degrees as arms were horizontally adducted, as described by Boettcher et al. [24]. Subjects held the reference MVIC for 5 sec, and the middle 3 sec were used for analysis. The mean of 3 normalization contractions was used for calculating percent MVIC. Subjects rested for 90 sec between each MVIC trial.

### 2.4. Functional Outcome Measurements

Subjects' functional ability was assessed using 5 standardized measurements. These evaluated function in several domains including balance, gait, strength, frailty, and health-related quality of life. Both performance-based and self-report functional outcomes were included. The outcome measures used in this study will be briefly described; all have established and acceptable reliability and validity.

#### 2.4.1. Four Square Step Test

The 4 square step test was administered using a grid constructed with 4 PVC pipes (2.5 cm diameter) that were 91 cm long and connected in the center at 90-degree angles. The grid was secured to the floor with tape so that it would not move. Subjects were instructed to stand in square 1 facing square 2 and then were asked to step with both feet into each square as quickly as possible, first clockwise and then counterclockwise. Subjects were instructed to face forward so they stepped forward, backward, and sideways to the right and left [26]. Time was recorded for 3 trials, and the fastest trial was used in data analysis. The 4 square step test is an indicator of dynamic standing balance and gait ability [27].

#### 2.4.2. Postural Stability

Postural stability was measured via force plates (Biodex Stability System, Biodex Medical Systems, Shirley, NY) interfaced with computer software (Version 3.1) that calculated center of gravity movement. Subjects were tested under 4 conditions: (1) standing eyes open solid surface, (2) standing eyes closed solid surface, (3) standing eyes open foam surface, and (4) standing eyes closed foam surface. Subjects were asked to remove their footwear and then to stand on the force platform in a comfortable stance with feet apart. Foot position was adjusted using visual input until the center of gravity was aligned with the center of the force plate. This process was repeated before each testing condition, and subjects did not move their feet between trials. Subjects were also instructed to keep their arms hanging at their sides and to only touch the handrails if needed to prevent a fall. Subjects performed 3 trials under each condition for 20 sec with a minimum of 10 sec of rest between trials. Stability index for overall, anterior-posterior, and medial-lateral was calculated from the center of gravity movement. Postural stability measurements indicated static standing general balance ability (overall), anterior-posterior sagittal plane balance ability, and medial-lateral frontal plane balance ability [28, 29]. Higher values represent greater center of gravity movement and less balance stability than lower values.

#### 2.4.3. Sit to Stand Test

A 30 sec sit to stand test was administered to subjects using an armless chair that was 46 (height) × 46 (width) × 46 (depth) cm. Subjects were instructed to sit in the middle of the chair, place their hands on opposite shoulders crossed at the wrist, keep feet flat on the floor, rise to a full standing position, sit back down, and repeat for 30 sec [30]. The number of complete sit to stand and stand to sit cycles was recorded. The sit to stand test is an indicator of lower body strength. This test has been used previously in research involving patients recovering from heart surgery [7, 31]. Sit to stand performance has also been shown to be associated with balance, disability, and frailty [32, 33].

#### 2.4.4. Handgrip Force

Handgrip strength was measured with a digital dynamometer (GripTrack Commander; JTECH Medical, Salt Lake City, UT). For all subjects, the handle of the dynamometer was set at the middle position. Subjects stood with their arms at their sides (shoulder in neutral, elbow in extension, and wrist in neutral), and 3 trials were recorded. Subjects were instructed to squeeze as hard as possible and hold for 2 sec and then relax. Subjects performed 3 trials with each hand. Rest as needed was provided between trials. The best of the 3 trials was used in data analysis. Handgrip strength is an indicator of overall upper body strength. Grip strength measured using a handgrip dynamometer is an objective measurement that has been shown to be significantly correlated with frailty and function in patients with vascular disease and patients recovering from cardiac surgery [34].

#### 2.4.5. RAND 36-Item Short Form Health Survey

**Figure 1:**
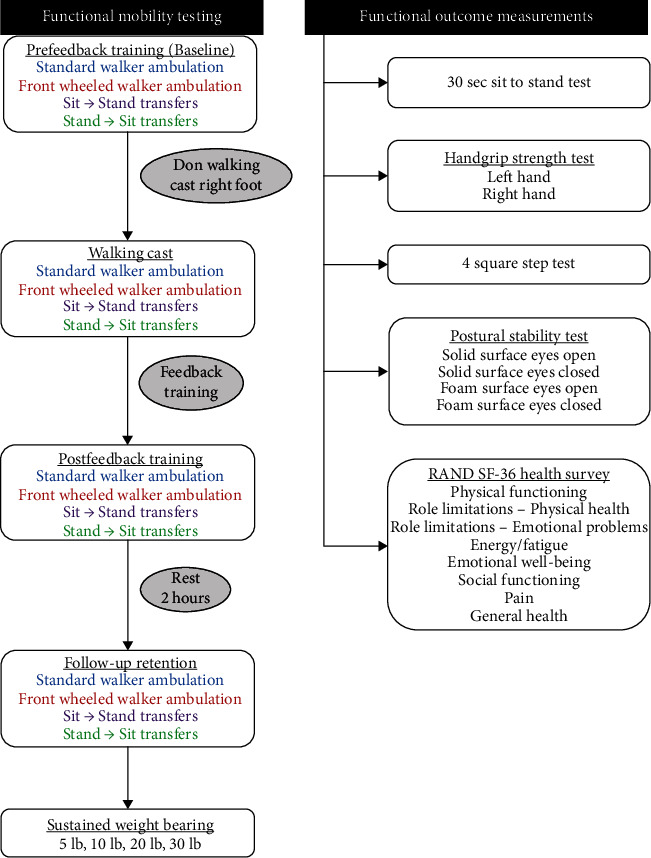
Study protocol flowchart and components.

In addition, the RAND 36-Item Short Form Health Survey (SF-36) was completed by all subjects. The SF-36 is a self-report measure of generic health-related quality of life and generates 8 subscale scores outlined in Figure 1. Scores on the SF-36 range from 0 to 100 with higher values representing a more positive health state than lower values [35]. The SF-36 has been widely used in research evaluating a variety of populations including community dwelling older adults [36] and patients recovering from heart surgery [37]. Previous studies have shown that SF-36 scores are related to functional ability and physical activity [7].

### 2.5. Procedures

After obtaining informed consent, subjects underwent a basic health history and physical examination to obtain baseline physiological data and information pertaining to their health. First, subjects completed an intake questionnaire to ensure they met study criteria and to collect demographic data. Next, a screening examination was completed which included resting vital signs, body mass index, upper extremity range of motion, strength, sensation, and coordination, gait, and balance. The Timed Up and Go Test was used as a safety screen for balance impairment. Subjects were instructed to stand up from a chair, walk to and around a cone 3 meters away, and return to sitting in the chair. A time of greater than 14 sec indicates increased risk of falling and was used as an exclusion criterion [38].

The testing procedures were explained to the study participant, and 2 bipolar EMG electrodes were placed on the participant's upper chest as described previously to measure PM muscle activity. Next, subjects performed the 3 MVIC that were averaged and used for data normalization. Data collection took place during 4 functional mobility tasks which included (1) ambulation using a standard walker, (2) ambulation using a front wheeled walker, (3) standing up from a chair, and (4) sitting down in a chair. For each functional mobility task testing took place under 4 different conditions including (1) prefeedback training (self-selected), (2) leg impairment simulated by wearing a walking cast, (3) postfeedback training, and (4) 2-hour follow-up. Study components are outlined in Figure 1. All trials of walking with an assistive device included a minimum of 5 steps, and all trials of transferring from a chair included 3 repetitions of the movement. Testing was stopped if a study participant experienced any pain or was unable to perform the task safely (i.e., with proper form, without loss of balance).

During both walker ambulation trials, subjects were instructed to put less than 10 pounds of pressure through each arm and to walk until instructed to stop. Subjects were allowed to determine which foot they initiated stepping with, and a digital marker was placed at each heel contact of that foot. The second through fourth gait cycles were used for data analysis. For EMG data analysis, the time capture window was set from the beginning of a heel strike to the beginning of the next heel strike.

During both of the transfer trials, subjects were instructed to put 5-10 lb of pressure through each arm while standing up and sitting down. During all trials, markers were placed at the initiation (hand on the dynamometer handles) and completion of the movement (hands off the dynamometer handles). For EMG data analysis, the time capture window was set from the beginning of the first marker to the end of the second marker.

After completing all 4 functional tasks using self-selected movement strategies (pretraining), subjects were fitted with a walking cast to simulate how a leg impairment could influence UE weight bearing during functional mobility. A stockinette (Rolyan Economy 4” Cotton Stockinette, model # 77432, Performance Health, Warrenville, IL) was placed on the right foot before an appropriately sized walking cast was applied. (Air Cam Walker Fracture Boot, Black, Small Model #USA14103, Medium model # USA14105, Large model # USA14107, Extra Large Model #USA14109, United Ortho, Fort Wayne, Indiana). Then, subjects performed the same 4 functional tasks wearing the walking cast while force and EMG data were recorded again.

Next, subjects were given an intervention using feedback training. The feedback protocol included 30 sec training sessions repeated once after a brief rest period as follows: (1) visual feedback standing in place putting approximately 10 lb of force through the instrumented walker, (2) auditory feedback (buzzer when force exceeding 10 lb) while ambulating with the standard and the front wheeled walker, (3) visual feedback sitting in place putting approximately 10 lb of force through the instrumented “chair” handles twice, and (4) auditory feedback (buzzer when force exceeded 10 lb) during sit to stand transfers. Subjects were tested again immediately after feedback training and then again 2 hours after finishing feedback training. Order of functional tasks during data collection was randomized, and subsequent testing took place in the same order.

Lastly, subjects completed 4 trials of sustained weight bearing through the instrumented walker. Using continuous visual feedback, they used constant pressure of 5 lb (2.3 kg), 10 lb (4.5 kg), 20 lb (9.1 kg), and 30 lb (13.6 kg) for 15 sec each. Both peak and average forces were recorded simultaneously with PM muscle EMG data. Lastly, subjects completed the 5 functional outcomes measures (see Figure 1) in random order.

### 2.6. Statistical Analyses

Mean EMG (of 3 trials) and peak force (over 3 trials) data were used in statistical testing. All EMG data were normalized and expressed as a percent of MVIC. Descriptive statistics for subject demographic variables, UE force, PM muscle EMG activity, and functional measurements were calculated. To determine differences in measurements among the functional and testing conditions, ANOVA and post hoc tests were used. Differences among the 4 functional tasks were determined using Tukey's Honest Significant Difference. Pair-wise comparisons between prefeedback training and walking boot, prefeedback training and postfeedback training, and postfeedback training were performed using *t*-tests and a Bonferroni correction. The alpha level was set at <0.05. Pearson product moment correlations were used to examine the relationship between PM muscle EMG activity and UE force and the relationship between functional and demographic variables and UE force. Statistical analyses were performed using Excel ToolPak (Microsoft Corporation, Redmond, WA).

## 3. Results

Subject baseline demographic information is outlined in Table 1. The participants (*n* = 30) in this study had a mean (±SD) age of 68.6 (±6.4) years and 53% were men. Their mean height, weight, and body mass index were 171.2 (±10.0) cm, 84.4 (±18.1) kg, and 28.2 (±5.8) kg/m^2^, respectively. Mean time to complete the Timed Up and Go was 7.3 (±1.4) seconds. Study participants' mean resting vital signs were as follows: heart rate 72 (±10) bpm, systolic blood pressure 126 (±16) mmHg, and diastolic blood pressure 77 (±7) mmHg. On average, subjects had 2.8 (±1.8) comorbidities reported which most commonly included chronic back pain (53%), arthritis (43%), previous fractures (40%), cardiovascular disease (33%), and osteoporosis (20%).

### 3.1. Force Data

**Figure 2:**
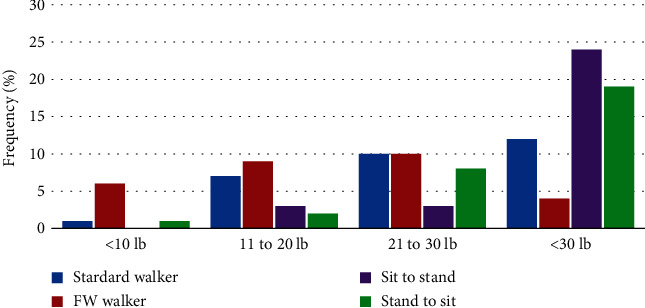
Frequency (%) of upper extremity force for all functional task trials. FW: front wheeled.

**Figure 3:**
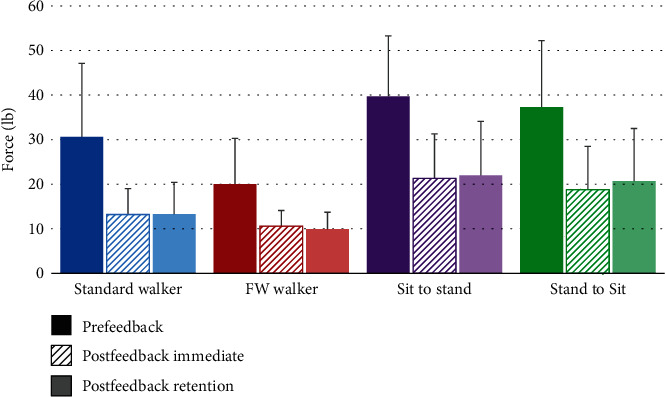
Force data (mean ± SD) before, immediately following, and 2 hours after feedback training.

Force data during each functional mobility task and testing condition are shown in Table 2. Prior to feedback training both with and without the walking cast, subjects used significantly less UE force during front wheeled walker ambulation than during the other functional tasks. For both postfeedback and follow-up retention, UE force was less during ambulation than transfers. Mean values during all tasks were greater than 10 lb (4.5 kg), the weight limit commonly prescribed with sternal precautions. During a majority of the trials (92%), study participants incorrectly overestimated and used too much UE force which is shown in Figure 2.  Figure 3 shows UE weight bearing force before, immediately following, and 2 hours after feedback training. After feedback training, UE force was significantly less than at baseline (prefeedback training). Two hours following feedback training, UE force was similar to that during immediate postfeedback for all 4 functional tasks.

**Figure 4:**
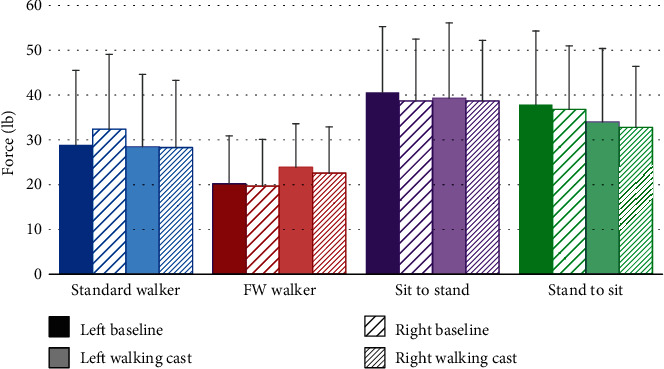
Force symmetry data (mean ± SD) with and without lower extremity impairment (wearing a walking cast).

Peak forces for both the left and the right UE are shown in Figure 4. The differences between right and left ranged from 0.2 to 3.5 lb (0.1 to 1.6 kg) of force. For both baseline and walking cast trials, there were no significant differences between the left and right sides. When comparing UE force between walking cast on vs. off, we found that UE force was only greater when subjects were wearing the cast than at baseline during standard walker ambulation.

### 3.2. EMG Data

**Figure 5:**
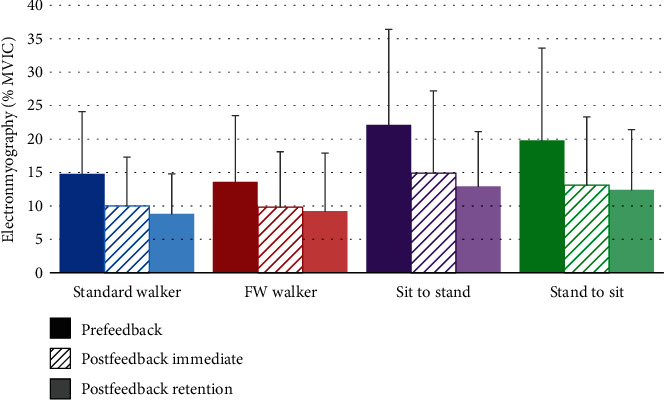
Pectoralis major electromyography data (mean ± SD) before, immediately following, and 2 hours after feedback training.

Muscle EMG data during each functional mobility task and testing condition are shown in Table 3. Prior to feedback training, PM muscle EMG activity was significantly less during front wheeled walker ambulation than during sit to stand transfers. While wearing the walking cast, PM muscle EMG activity was significantly less during walker ambulation than during sit to stand transfers. During all functional tasks, there were no significant differences between PM muscle EMG activity wearing a walking cast compared to without it (prefeedback training).  Figure 5 shows PM muscle EMG before, immediately following, and 2 hours after feedback training. After feedback training (both immediately and at 2 hours follow-up), muscle EMG was significantly less than at baseline (prefeedback training) for all 4 functional tasks.

### 3.3. Functional Outcome Measurement Data

Table 4 outlines the descriptive statistics of the functional outcome measures used in this study. Correlations among functional outcome measurement scores and force measurements during all 4 functional tasks at baseline (prefeedback training) are shown in Table 5. The highest correlations (*r* = 0.56) were found between handgrip strength and transfers. Moderate correlations (*r* = 025‐0.50) were found between walker ambulation and handgrip strength, 4 square step test, SF-36 role limitations, SF-36 social functioning and also between sit-stand transfers and center of gravity anterior-posterior stability index, SF-36 emotional problems, and body mass index.

### 3.4. Correlational Data

Intersubject correlations between PM muscle EMG values and average UE force ranged from 0.10 to -0.16, and peak UE force ranged from 0.08 to 0.29 for the study participants. Intrasubject correlations between PM muscle EMG values and peak UE force ranged from 0.47 to 0.97 as shown in Table 6. Intrasubject correlations between PM muscle EMG values and average UE force ranged from 0.50 to 0.99 as shown in Table 7.

## 4. Discussion

This study revealed that during all of the functional tasks performed before feedback training, on average, the force put through the UE by the study participants exceeded that generally recommended with sternal precautions 10 lb (4.5 kg) or less [5, 8, 9]. With only verbal instructions regarding UE weight bearing, patients may not be able to accurately estimate the amount of force put through their UE. Po-Chen and Cherng [39] found that healthy older adults on average put 22-28 lb (10.0-12.7 kg) of UE force through a standard walker when given no instructions to limit weight which is similar to the mean value during ambulation with a standard walker in our study of 30.6 lb (13.9 kg). Ishikura [40] found that when healthy subjects used a 4 wheeled walker, force put through the UE varied from 13 to 40% of body weight during a gait cycle which is similar in magnitude to our data for front wheeled walker ambulation (on average up to 25% of body weight).

This same trend has been observed in several previous studies that examined weight bearing through the lower extremities and, therefore, inversely through walker handles, during ambulation in patient populations. Fast and colleagues [41] found that when patients used a standard walker to reduce weight bearing through the lower extremities, force through the walker was 20-49 lb (9.1-12.7 kg), but when used for balance, the force through the walker was only 2-28 lb (0.9-12.7). Because patients recovering from cardiac surgery with median sternotomy typically do not need to unweight the lower extremities, and use a walker primarily for balance stability, the latter values reported by Fast et al. are similar to the mean of 30.6 lb (13.9 kg) found in our current study. However, it should be noted that in the studies by Po-Chen and Cherng [39], Ishikura [40], and Fast et al. [41], force was measured through a walker without instructions to limit UE weight bearing, and the studies were focused on limiting lower extremity weight bearing. In a cohort of young subjects, a previous study found that average UE weight bearing during ambulation with a walker was greater than 10 lb (4.5 kg) [22].

Studies examining compliance with touch down weight bearing (defined as <25 lb) in patients with lower extremity trauma have found that placing too much weight through the involved side is common [42, 43]. Hustedt and colleagues instructed healthy subjects to use touch down weight bearing with axillary crutches and found that weight was often up 2.5 times greater than the prescribed restriction [44, 45]. Similarly, we found that subjects commonly applied too much UE weight bearing force through a walker when given no feedback, and they often exceeded the 10 lb precautionary limit by 2-3-folds.

Ambulation using a front wheeled walker produced less UE weight bearing force than ambulation using a standard walker, which is consistent with a previous study that found that when young subjects were asked to place less than 10 pounds of pressure through walker handles, greater UE weight bearing force occurred when ambulating with a standard walker (18.5 lb) compared to a front wheeled walker (11.7 lb) [22]. This finding supports current sternal precautions and general clinical consensus that if an assistive device is needed for ambulation, a front wheeled walker is preferable for patients after median sternotomy [7, 9]. A plausible explanation is that patients using a standard walker must lift it off the ground for a portion of the gait cycle and then place it back down, whereas when using a front wheeled walker it remains on the ground throughout the gait cycle. Lifting a walker may result in less consistent force through the walker, creating cyclical peaks above the 10 lb (4.5 kg) limit. Previous studies have shown that lower extremity weight bearing (and therefore inversely UE weight bearing) varied throughout the gait cycle during ambulation with a 4 wheeled walker and standard walker [40, 41, 46]. Another possible explanation for our finding that less UE weight was placed through the front wheeled walker as compared to the standard walker relates to the type of gait pattern employed. When patients ambulate using a standard walker they use a “step-to” pattern, but they use a “step-through” pattern with a front wheeled walker, so UE force is distributed more evenly throughout the gait cycle and which helps reduce peak forces.

Results indicate that UE weight bearing force was lower during walker ambulation than sit-stand transfers at baseline and with a simulated lower extremity impairment (walking boot). Although studies have examined UE forces during activities of daily living [20, 21], little information is available on UE force during transfers. In previous studies, Anglin and Wyss [47] and LaPier and Cleary [22] reported higher maximal UE loads during stand to sit than during sit to stand which is not consistent with the results of this study. This may be related to the older age of the cohort in our current study. Perhaps older adults need more UE assistance with the concentric sit to stand portion of transfers than younger adults.

The results of this study demonstrate that a brief feedback intervention can be effective at reducing UE force exerted during both ambulation with an assistive device and sit-stand transfers. Both visual and auditory feedback were utilized to provide participants information regarding force placed through their UE. A recent systematic review examining the efficacy of feedback for improving gait parameters found that visual (60%) and auditory (40%) feedback were most commonly employed [48]. In the current study, a combination of both auditory (buzzer) and visual (force output on tablet screen) feedback was given to subjects. This feedback was provided concurrently (during practice of the skill) as opposed to terminally (after practice of the skill) to best facilitate acquisition of a novel skill. Study participants were first given prescriptive (information on exactly how much weight was being placed through the walker) visual feedback while statically practicing UE weight bearing in standing or sitting. Then, they were given descriptive (buzzer was sounded if force exceeded the 10 lb limit) auditory feedback while practicing the whole task [48]. This method facilitated part practice first followed by whole practice, a commonly employed principle of motor learning [49]. After feedback training, UE force was reduced during all functional tasks by 46-56%. But the average of each functional task was still greater than the 10 lb (4.5 kg) limit typically prescribed with sternal precautions. Although other studies [22, 42, 44, 45] have demonstrated that concurrent visual feedback training is effective at modulating force when weight bearing is limited, study results suggest that a more intensive feedback intervention (i.e., more repetitions of practice within a training session) and/or multiple training sessions would be ideal for older patients recovering from median sternotomy.

Results of the study also demonstrated that the immediate improvements in UE weight bearing force are retained in the short term. Force during all functional tasks was not significantly different immediately postfeedback training or/and with repeated testing 2 hours later. Hustedt and colleagues [50] also found 2-4 hour retention of lower extremity touch down weight bearing compliance after feedback training. In addition, they also found no significant differences in lower extremity force immediately after feedback training, 6-8 hours later, and 22-24 hours later, suggesting that retention of a single bout of feedback training may last up to 24 hours [50].

Results of this study suggest that PM muscle activation is relatively small (<23% MVIC) during functional mobility and that feedback training can further minimize it. During this study, PM muscle activity was measured because it attaches to the lateral borders of the sternum, pulls horizontally from medial to lateral, and is the primary mover for shoulder horizontal adduction. To date, only a single study has examined PM muscle EMG activity during ambulation with a walker or during sit-stand transfers [22]. A few studies have measured PM muscle EMG activity during similar activities. Pectoralis major muscle EMG activity was 13.0-14.4% MVIC while pushing a 4 kg weight forward on tracks and 7% MVIC in the “prayer” position (kneeling with hands on floor in front of knees) both of which are different shoulder positions than used in this study [51]. A previous study [22] found lower PM muscle activation in a cohort of young subjects which ranged from 3.0% to 9.2% as compared to our findings in older subjects which ranged from 13.6% to 22.1%.

This study revealed interesting relationships between function mobility and peak UE weight bearing force. Surprisingly, lower extremity strength (30 sec sit to stand test) was not correlated with the amount of UE weight bearing during the functional tasks, and handgrip strength was directly associated with UE weight bearing force during transfers and standard walker ambulation. Other studies have found that handgrip strength is an important prognostic indicator of frailty, which Graham and Brown [10] defined as “a geriatric syndrome characterized by an excess vulnerability to stressors, with reduced ability to maintain or regain homeostasis after a destabilizing event” that is associated with “an increased risk of disability, morbidity, and mortality.” There were moderate correlations between UE weight bearing force and balance and during walker ambulation (4 square step test) and sit-stand transfers (center of gravity anterior-posterior stability index). In addition, quality of life was also related to UE weight bearing force during functional mobility tasks, specifically the subdomains of role limitations—physical health, role limitations—emotional, and social functioning. When examining demographic characteristics (age, height, weight, and body mass index), only body mass index had a moderate relationship with peak UE weight bearing force during sit-stand transfers. In summary, 4 square step test, SF-36, and body mass index could be helpful clinically to identify patients susceptible to using UE weight bearing force greater than 10 lb (4.5 kg) during functional mobility tasks and, therefore, determine who would benefit most from postoperative feedback training with an instrumental walker.

Study results demonstrated that the relationship between PM muscle and UE weight bearing force is strong within subjects. In a previous study, we found strong intrasubject correlations between PM muscle EMG values and both peak and average UE force [22]. Muscle activation in this study was monitored via EMG, which measures the degree of muscle activation (motor neuron recruitment) by quantifying the number of action potentials; as such, EMG is an indirect indicator of muscle force production. Intrasubject correlations were strong (most >75%) suggesting that estimating UE force using PM muscle EMG values for a given subject might be possible.

Overall, our findings suggest that use of an instrumented walker and feedback training would be beneficial in clinical practice, especially to help older patients more accurately follow weight bearing instructions. Most previous studies have used bathroom scales [44, 45], force plates/pressure sensing mats [40, 47], or foot pressure sensors [42, 44, 46] to measure weight bearing through the lower extremities. Others have described use of an instrumented walker to measure force placed through the assistive device, but commonly, this was accomplished with sensors placed in the walker legs and not the handles [39, 41]. The instrumented walker used in this study had force transducers incorporated into the handles which Khodadadi et al. found result in easier installation and less error compared to installation on circular vertical walker legs [52]. An inexpensive, lightweight walker without bulky add-ons for the measurement and display of force would have multiple clinical applications for providing feedback to patients who need to limit weight bearing through the upper or lower extremities. In addition to patients recovering from median sternotomy, an instrumented walker and feedback training would be useful for patients who need to limit weight through a lower extremity, for example, following a fracture [42, 43]. In this study, weight bearing was equal bilaterally through the UE, indicating that possibly only a single walker handle needs to be instrumented. Although, UE force asymmetry may occur in patients with significant unilateral extremity disorders.

Several limitations need to be considered when generalizing the results of this study. This prospective study only included 30 subjects, but based on effect size, this provided adequate statistical power (>80%). Older (60-85 years of age) healthy study participants were utilized in order to evaluate the safety of the instrumented walker and to reduce variability due to other factors, such as pain and impaired cognition that can occur after surgery. It would be expected that this functionally independent population should be better able to use only 10 lb (4.5 kg) or less of UE weight bearing force than populations with functional limitations and/or that use an assistive device for mobility. Leung and Yeh [53] found that during sit-stand transfers using a walker, older adults who did not ambulate with a walker used less vertical UE force than those who did ambulate with a walker. In addition, force through the UE does not necessarily equal force across the sternum. McQuade and colleagues [46] found that although patients put 46% of their body weight through a walker during ambulation, compressive forces at the UE joints were only 20% of body weight, were greatest at the wrist, and decreased proximally. Lastly, this study found benefits of feedback training on modulation of weight bearing immediately and 2 hours after feedback training. Therefore, conclusions about long-term retention (>2 hours) cannot be made. Hustedt and colleagues [50] found improvements in lower extremity weight bearing were maintained up to 24 hours following feedback training, although others have not found good retention of acquired skills after feedback training [54].

In conclusion, the results of this study suggest that older patients may not be good at estimating UE force during weight bearing activities. Specifically, older patients recovering from median sternotomy most likely are not limiting arm force to 10 lb (4.5 kg) or less during daily activities, which puts them at risk for delayed bone healing and complications. However, a combination of visual and auditory feedback was effective at reducing UE weight bearing and PM muscle EMG activity. Study results suggest that balance, quality of life, and BMI may be important prognostic indicators of frailty in patients recovering from open heart surgery who may have difficulty limiting UE force to <10 lb (4.5 kg). Objective feedback training while using an assistive device would be useful for older patients recovering not only from median sternotomy but also from lower extremity fractures and/or surgeries that require limiting weight placed through a leg.

## Figures and Tables

**Table 1:** Subject descriptive demographic data.

Characteristic	Mean ± SD or frequency
Gender (male)	53%
Age (years)	68.6 ± 6.4
Height (cm)	171.2 ± 10.0
Weight (kg)	84.4 ± 18.1
Body mass index (kg/m^2^)	28.2 ± 5.8
Timed up and go (sec)	7.3 ± 1.4
Heart rate (bpm)	72 ± 10
Systolic blood pressure (mmHg)	126 ± 16
Diastolic blood pressure (mmHg)	77 ± 7
Comorbidities (total)	2.8 ± 1.8
Cardiovascular disease	33%
Diabetes	3%
Lung disease	10%
Kidney disease	7%
Cancer history	13%
Arthritis	43%
Osteoporosis	20%
Previous fracture	40%
Chronic Back pain	53%
Other	20%

**Table 2:** Force (lb) descriptive data (mean ± SD and range) for functional tasks prefeedback training, wearing a walking boot, postfeedback training, and follow-up retention at 2 hours.

	Prefeedback training	Walking boot	Postfeedback training	Follow-up retention
Standard walker	30.6 ± 16.5 (9.6–72.0)	28.4 ± 15.4 (6.9–69.3)	13.2 ± 5.8^+^ (5.4–25.0)	13.3 ± 7.1^+^ (5.4–35.2)
Front wheeled walker	20.0 ± 10.3^#^ (3.2 + 45.8)	23.2 ± 9.6^#+^ (5.4–46.4)	10.6 ± 3.5^+^ (5.0–18.7)	9.9 ± 3.8^+^ (3.0–21.7)
Sit to stand	39.7 ± 13.6^∗^ (14.2–68.4)	39.0 ± 14.8^∗^ (10.8 + 75.9)	21.3 ± 10.0^#^^∗^^+^ (7.6–49.8)	22.0 ± 12.1^#^^∗^^+^ (4.0–54.1)
Stand to sit	37.3 ± 14.9^∗^ (8.8–64.8)	33.4 ± 14.5^∗^^+^ (9.3–64.2)	18.8 ± 9.7^#^^∗^^+^ (3.0–51.0)	20.7 ± 11.8^#^^∗^^+^ (4.9–51.3)

^#^Significantly different from standard walker; ^∗^significantly different from front wheeled walker; ^+^significantly different from prefeedback training.

**Table 3:** Pectoralis major muscle electromyographic (% maximal voluntary isometric contraction) descriptive data (mean ± SD and range) for functional tasks prefeedback training, wearing a walking boot, postfeedback training, and follow-up retention at 2 hours.

	Prefeedback training	Walking boot	Postfeedback training	Follow-up retention
Standard walker	14.8 ± 9.3% (3.9–36.8%)	14.5 ± 9.2% (4.5–40.7%)	10.0 ± 7.3%^+^ (2.9–26.8%)	8.8 ± 6.0% (2.3–21.0%)
Front wheeled walker	13.6 ± 9.9% (2.8 + 38.3%)	16.0 ± 10.1% (4.1–34.3%)	9.8 ± 8.3%^+^ (1.5–32.3%)	9.2 ± 8.7% (1.1–32.3%)
Sit to stand	22.1 ± 14.3%^∗^ (6.2–67.0%)	24.2 ± 12.0%^#^^∗^ (8.7–56.5%)	14.9 ± 12.3%^+^ (1.7–51.9%)	12.9 ± 8.2% (1.9–31.6%)
Stand to sit	19.8 ± 13.8% (3.7–64.2%)	19.0 ± 11.5% (6.0–52.6%)	13.1 ± 10.2%^+^ (1.8–44.8%)	12.4 ± 9.0% (3.0–32.4%)

^#^Significantly different from standard walker; ^∗^significantly different from front wheeled walker; ^+^significantly different from prefeedback training.

**Table 4:** Functional outcome measurement descriptive data (mean ± SD and range).

Functional outcome measurement	Mean ± SD	Range
30 sec sit to stand test (reps)	12.5 ± 3.1	8–20
Handgrip (lb)	68.2 ± 25.1	28.5–128.5
4 square step test (sec)	9.9 ± 1.7	5.8–13.3
Postural sway solid surface—eyes open		
COG overall stability index	2.78 ± 1.47	0.73–6.79
COG anterior-posterior stability index	1.97 ± 1.53	0.38–6.52
COG medial-lateral stability index	1.57 ± 0.93	0.16–3.85
Postural sway solid surface—eyes closed		
COG overall stability index	3.55 ± 1.63	1.08–7.68
COG anterior-posterior stability index	2.47 ± 1.73	0.65–7.21
COG medial-lateral stability index	2.03 ± 1.17	0.33–5.6
Postural sway foam surface—eyes open		
COG overall stability index	2.83 ± 1.30	0.90–6.33
COG anterior-posterior stability index	1.85 ± 1.20	0.56–4.75
COG medial-lateral stability index	1.7 ± 1.1	0.5–6.0
Postural sway foam surface—eyes closed		
COG overall stability index	4.8 ± 1.4	3.3–8.1
COG anterior-posterior stability index	3.3 ± 1.2	1.4–6.1
COG medial-lateral stability index	2.7 ± 1.1	1.0–6.3
Quality of life (SF-36)		
Physical functioning	82.7 ± 12.1	55–100
Role limitations—physical health	78.3 ± 33.9	0–100
Role limitations—emotional problems	93.4 ± 16.1	33–100
Energy/fatigue	69.5 ± 18.5	30–100
Emotional well-being	79.3 ± 21.6	0–100
Social functioning	94.0 ± 11.8	50–100
Pain	77.9 ± 16.5	35–100
General health	76.5 ± 18.8	15–100

COG: center of gravity; SF-36: RAND 36-Item Short Form Health Survey.

**Table 5:** Correlations between peak force and functional outcome measurement scores.

	Standard walker	Front wheeled walker	Sit to stand	Stand to sit
*Functional outcomes*				
30 sec sit to stand test	-0.14	0.09	-0.11	-0.09
Handgrip	0.35	0.03	0.56	0.56
4 square step test	-0.33	-0.35	-0.04	-0.04
COG overall stability index	0.20	0.22	0.25	0.19
COG anterior-posterior stability index	0.21	0.23	0.31	0.32
COG medial-lateral stability index	0.04	0.05	0.08	-0.03
*Quality of life (SF-36)*				
Physical functioning	0.13	0.23	0.05	0.09
Role limitations—physical health	-0.32	-0.04	-0.22	-0.12
Role limitations—emotional problems	-0.33	-0.29	-0.31	-0.30
Energy/fatigue	-0.22	-0.16	-0.08	-0.14
Emotional well-being	0.03	-0.10	0.10	-0.02
Social functioning	-0.38	-0.38	-0.24	-0.24
Pain	-0.02	0.00	0.03	0.09
General health	-0.22	-0.20	-0.23	-0.22
*Demographic characteristics*				
Age (years)	-0.13	-0.02	-0.02	-0.01
Height (cm)	-0.13	-0.02	-0.02	-0.01
Weight (kg)	-0.13	-0.02	-0.02	-0.01
Body mass index (kg/m^2^)	0.17	0.16	0.27	0.35

COG: center of gravity; SF-36: RAND 36-Item Short Form Health Survey.

**Table 6:** Intrasubject correlations between peak force and pectoralis major muscle electromyography data.

Subject #	*r*	Subject #	*r*	Subject #	*r*
1	0.55	10	0.93	19	0.93
2	0.81	11	0.78	20	0.93
3	0.97	12	0.95	21	0.96
4	0.77	13	0.93	22	0.91
5	0.91	14	0.84	23	0.65
6	0.83	15	0.82	24	0.51
7	0.89	16	0.77	25	0.64
8	0.97	17	0.47	26	0.66
9	0.85	18	0.67	27	0.55

**Table 7:** Intrasubject correlations between average force and pectoralis major muscle electromyography data.

Subject #	*r*	Subject #	*r*	Subject #	*r*
1	0.50	10	0.93	19	0.90
2	0.64	11	0.76	20	0.96
3	0.96	12	0.95	21	0.94
4	0.58	13	0.90	22	0.96
5	0.91	14	0.84	23	0.69
6	0.81	15	0.79	24	0.63
7	0.87	16	0.59	25	0.64
8	0.96	17	0.64	26	0.67
9	0.86	18	0.77	27	0.50

## Data Availability

The data that support the findings of this study are available from the corresponding author upon request.
